# Post-harvest cleaning, sanitization, and microbial monitoring of soilless nutrient delivery systems for sustainable space crop production

**DOI:** 10.3389/fpls.2024.1308150

**Published:** 2024-10-11

**Authors:** Aaron B. Curry, Cory J. Spern, Christina L. M. Khodadad, Mary E. Hummerick, LaShelle E. Spencer, Jacob Torres, J. Riley Finn, Jennifer L. Gooden, Oscar Monje

**Affiliations:** ^1^ Engineering Research and Consulting LLC, Kennedy Space Center, Merritt Island, FL, United States; ^2^ Noetic Strategies Inc., Kennedy Space Center, Merritt Island, FL, United States; ^3^ Amentum, Kennedy Space Center, Merritt Island, FL, United States; ^4^ The Bionetics Corporation, Kennedy Space Center, Merritt Island, FL, United States; ^5^ Aetos Systems Inc., Kennedy Space, Merritt Island, FL, United States; ^6^ Air Revitalization Lab, Aetos Systems Inc., Kennedy Space Center, Merritt Island, FL, United States

**Keywords:** spaceflight, bioregenerative food system, food safety, sanitization, microbial monitoring, exploration, sustainable, space crop production

## Abstract

Bioregenerative food systems that routinely produce fresh, safe-to-eat crops onboard spacecraft can supplement the nutrition and variety of shelf-stable spaceflight food systems for use during future exploration missions (i.e., low earth orbit, Mars transit, lunar, and Martian habitats). However, current space crop production systems are not yet sustainable because they primarily utilize consumable granular media and, to date, operate like single crop cycle, space biology experiments where root modules are sanitized prior to launch and discarded after each grow-out. Moreover, real-time detection of the cleanliness of crops produced in spacecraft is not possible. A significant paradigm shift is needed in the design of future space crop production systems, as they transition from operating as single grow-out space biology experiments to becoming sustainable over multiple cropping cycles. Soilless nutrient delivery systems have been used to demonstrate post-harvest sanitization and inflight microbial monitoring technologies to enable sequential cropping cycles in spacecraft. Post-harvest cleaning and sanitization prevent the buildup of biofilms and ensure a favorable environment for seedling establishment of the next crop. Inflight microbial monitoring of food and watering systems ensures food safety in spaceflight food systems. A sanitization protocol, heat sterilization at 60°C for 1 h, and soaking for 12 h in 1% hydrogen peroxide, developed in this study, was compared against a standard hydroponic sanitization protocol during five consecutive crop cycles. Each cropping cycle included protocols for the cultivation of a crop to maturity, followed by post-harvest cleaning and inflight microbial monitoring. Microbial sampling of nutrient solution reservoirs, root modules, and plants demonstrated that the sanitization protocol could be used to grow safe-to-eat produce during multiple crop cycles. The cleanliness of the reservoir and root module surfaces measured with aerobic plate counts was verified in near real time using a qPCR-based inflight microbial monitoring protocol. Post-harvest sanitization and inflight microbial monitoring are expected to significantly transform the design of sustainable bioregenerative food and life support systems for future exploration missions beyond low earth orbit (LEO).

## 1 Introduction

The ability to conduct long-term exploration missions beyond LEO (i.e., Gateway and Mars-transit) and establish sustainable, Earth-independent colonies on the Moon and Mars will require reliable bioregenerative plant growth technologies to supplement spaceflight food systems with fresh crops. Space farm modules are built as flight hardware for feeding crews in space using Earth-based agricultural practices and crops adapted to grow optimally in new environments with new biophysical combinations (e.g., microgravity, supra-elevated CO_2_ concentration, low atmospheric pressure, and deep space radiation) encountered only in space (Monje et al., 2003). Decades of spaceflight plant growth studies have demonstrated that plants in space grow at the same rate as on Earth (Ward et al., 1970; Bugbee and Salisbury, 1988; Monje et al., 2005; Paul et al., 2013; Zabel et al., 2016; Monje et al., 2020), but more work is needed to demonstrate that space crop production can be a reliable component of food and life support systems.

### 1.1 Spaceflight food systems

Sustainable bioregenerative food systems, designed for deployment in microgravity and deep-space radiation environments, must be developed to validate crop production technologies, and their food safety must be demonstrated prior to integration into exploration vehicle designs (Anderson et al., 2017). Important gaps, such as system reliability, establishment of microbiological standards for fresh produce, food safety protocols for consuming produce grown in spacecraft, inflight plant health monitoring, and microbiological testing methods for verifying food safety in spaceflight, must also be addressed before crop production systems can become integral components of spaceflight food systems (Anderson et al., 2017; Douglas et al., 2021; Poulet et al., 2022; Qin et al., 2023). Foodborne illness in spaceflight must be prevented because its effects on crew health can be more severe in spacecraft due to limited access to medical capabilities and supplies (Douglas et al., 2021; Lee et al., 2023). During Biosphere 2, decreased crop productivity related to unexpected reductions in light levels during the winter months caused the crew to experience weight loss and calorie restrictions (Silverstone and Nelson, 1996; Walford et al., 2002). Thus, even a partial loss of the food system due to poor crop growth or system malfunction may lead to an increased risk of food scarcity and decreased nutritional input (Douglas et al., 2021).

The shelf-stable spaceflight food system at the International Space Station (ISS) was tested to meet rigorous microbiological safety standards prior to launch. However, current food safety evaluation methods are not resource-efficient and cannot be conducted during spaceflight. Microbiological analyses of spaceflight samples from edible plants grown in the Russian Lada chamber on ISS (Hummerick et al., 2010, 2011) and from the Veggie facility on ISS (Khodadad et al., 2020; Hummerick et al., 2021) were conducted using culture-dependent methods that require samples to be returned to Earth for analysis. Microbiological analysis of frozen plant samples and root modules returned to Earth revealed that leaves and roots were colonized during growth on ISS by microbes found in water, surface, and air samples of the spacecraft (Khodadad et al., 2020; Hummerick et al., 2021; Lee et al., 2023). Although these data are useful for cataloging the organisms found on fresh crops in space and for generating relevant spaceflight tests and standards, they were not available to the crew in near real time. Anderson et al. (2017) suggested that inflight DNA sequencing and polymerase chain reaction (PCR) molecular techniques could be used to assess microbial loads on food products during spaceflight (Khodadad et al., 2021). Eventually, spaceflight microbial monitoring methods must progress beyond ground-based cultures of potentially harmful microorganisms toward culture-independent, swab-to-sequencer processes (i.e., using miniPCR and MinION) to be conducted in near real time (Stahl-Rommel et al., 2021; Poulet et al., 2022; Lee et al., 2023; Siegel et al., 2023). Currently, Veggie-grown crops consumed on ISS have been surface-sanitized using food safe disinfectant wipes (Massa et al., 2017) and similar protocols have been used to consume chili peppers grown in APH.

### 1.2 Sustainable space crop production

A significant paradigm shift is needed in the design of future space crop production systems, as they transition from operating as single grow-out space biology experiments to becoming sustainable during multiple cropping cycles. Thus, space crop production systems must be designed for repeated startup, shutdown, and dormancy and must perform sustainably and reliably during multiple cropping cycles consisting of planting, harvesting, and post-harvest cleaning operations with minimal resupply from Earth. Furthermore, the rooting area and mechanical design of their water and nutrient delivery subsystems (WNDS) must allow sanitization because system hygiene affects food safety and system reliability. This continuous cropping approach differs from space biology experiments, which often have single-use root modules that are sanitized prior to use but are considered a waste product after a single crop cycle.

Currently, the Veggie and Advanced Plant Habitat (APH) crop production facilities on the ISS utilize pre-planted root modules filled with porous substrates (e.g., 1 mm–2 mm arcillite) and slow-release fertilizer pellets (Massa et al., 2017; Monje et al., 2020). The root modules were packed in a laminar flow bench using sanitized seeds and autoclaved components (substrate media and wicks) to ensure a healthy environment for plants during germination and seed establishment. Veggie uses a passive watering system, whereas APH uses active moisture control, and their root modules are not reusable after harvest. This resulted in considerable mass allocation to the root modules. Veggie uses six plant growth pillows, each containing 210 g of arcillite, while APH houses four quadrants containing 1 kg arcillite/quadrant, corresponding to a resupply of 1.3 kg and 4 kg of single-use granular porous media, respectively, for each new crop cycle (Monje et al., 2019). New root modules are utilized for each crop cycle because the fertilizer becomes depleted after one grow-out, and because microbial growth and decay of roots from a previous crop can compete for O_2_ with germinating seedlings from the next crop. Studies in which seeds were replanted in used root modules showed that crop yield decreased in successive plantings due to nutritional deficiencies and the buildup of decomposing roots from previous plantings (Levinskikh et al., 2001; Stutte et al., 2009). Thus, the baseline configurations of the Veggie and APH facilities are not sustainable for long-duration missions because of the logistics of resupplying preplanted root modules for conducting single-crop experiments.

A key difference between terrestrial systems and current space crop production systems is the potential for terrestrial systems to have key components removed and cleaned separately, whereas space crop production systems require crew-friendly protocols for cleaning them in place. At present, the sanitization of Veggie and APH consists of post-harvest disinfection of chamber surfaces with disposable wipes containing a generally regarded as safe (GRAS) organic acid sanitizer (ProSan, Microcide, Sterling Heights, MI, USA). This minimal effort is effective because most of the microbial and decaying root biomass is discarded in the used root modules. In contrast, future crop production systems undergoing multiple cropping cycles will require periodic cleaning and sanitization to eliminate the risks from bacterial and fungal proliferation to the crew and hardware. Sanitization of the growth system after each cropping cycle prevents the buildup of biofilms, which may harm the watering system, and ensures food safety (Mogren et al., 2018). Post-harvest sanitization also contributes to a favorable environment for seed germination and seedling establishment in the next crop.

Sanitization agents for use in crop production systems onboard spacecraft must be compatible with life support equipment, human toxicity, and plant health. Thus, sanitization system designs must consider the storage, containment, and overall toxicity of consumables needed for sustained crop production. Commercial food production systems employ hydrogen peroxide, aqueous ozone, and other chemical agents such as quaternary ammonium compounds and sodium hypochlorite to sanitize and disinfect components of plant growth systems. However, chemicals used in crop production subsystems (e.g., fertilizer salts, acids/bases for pH control, bleach, or hydrogen peroxide for sanitization) must be carefully selected because they pose toxicological risks that threaten human health during spaceflight operations. Although bleach and quaternary ammonium compounds can be effectively removed from terrestrial food production systems via flushing, they can have detrimental effects if they accumulate within watering systems over time. One of the most widely used quaternary ammonium compounds, benzalkonium chloride, is a major irritant with adverse effects on the skin, eyes, and the inner ear (Baudouin et al., 2010; Aursnes, 1982). It has also been shown to inhibit the growth of crops that have been successfully grown in spaceflight, such as lettuce (Khan et al., 2018). These health concerns and excessive crew involvement in the addition and removal of harsh chemical sanitizers effectively eliminated them from consideration in this study.

Recently, a non-toxic 5% ProSan biocide was tested during ground studies to clean the water condensate recovery and root distribution reservoirs of APH (Monje, 2019). ProSan, a mixture of citric acid and a surfactant, was selected from numerous candidate sanitization agents for use in spacecraft because of its known sanitization efficacy, low payload chemical toxicity hazard level (THL), and material compatibility. In a study performed on plant material harvested from Orbitec Biomass Production System for Education (BPSe) plant chambers during a Veggie plant growth experiment, ProSan effectively reduced the total heterotrophic plate, yeast, and mold plate counts of Outredgeous lettuce and Mizuna mustard by an average of two orders of magnitude (Hummerick et al., 2012). Further microbial sampling studies determined that this biocide would be effective in sanitizing the APH waterline and reservoir subsystems. During ground testing in the APH, however, 5% ProSan acidified the water in the APH reservoirs to pH 3, and the added surfactants required 30 L–40 L of water to flush the system completely. Although ProSan is an effective, low-toxicity sanitizer, this sanitization scheme was found to be extremely reliant on crew time and resources and was not considered in this study.

Disinfectants produced in sanitization systems employing onboard sources (e.g., *in situ* generation of hydrogen peroxide, aqueous ozone, or plasma water) were evaluated in this study because they could reduce the dependence on ground-based resupply (Vijapur et al., 2022). These sanitizers are strong antimicrobial agents that prevent biofilm formation, exhibit relatively short lifetimes after being generated, and eventually degrade into water and oxygen (Megahed, 2018; Mogren et al., 2018; Zheng et al., 2020), making them compatible with spacecraft trace contaminant removal systems (Siegel et al., 2023). Vijapur et al. (2019, 2022) developed an *in situ* hydrogen peroxide generator that could simplify the logistics of supplying hydrogen peroxide, a THL-1 compound, for sanitization in space. Aqueous ozone is a strong oxidizer but is not corrosive and does not harm the skin. It can also be generated *in situ* at any scale using handheld or commercial units. Knowing the cleaning efficiency of sanitizers that can be generated *in situ*, such as hydrogen peroxide or aqueous ozone, can be used to determine which is more effective in the extraterrestrial environment.

In this study, we developed novel protocols for the system sanitization and inflight microbial monitoring of bioregenerative food systems to be deployed in spacecraft. This study was completed in two parts: 1) the efficacy of candidate *in situ*-generated sanitizers against bacteria known to form biofilms and bacterial spores was evaluated, and 2) a sanitization protocol was selected and its efficacy was compared to a control sanitization protocol across multiple crop cycles. Five consecutive 28-day crop cycles were conducted in three soilless systems: a porous tube-based WNDS (PTNDS), foam-based On-Demand WNDS (OD), and control hydroponic system using the nutrient film technique (NFT). Each crop cycle consisted of nutrient solution filling, followed by seed planting, thinning of seedlings, adjusting variable plant density during crop development, harvest, and post-harvest sanitization. The cleaning efficiency of the sanitization protocol used in the PTNDS and OD WNDS was compared to that of the hydroponic control by measuring the microbial and fungal loads from swab samples collected from nutrient reservoirs, root module surfaces, and edible plants. Microbial loads from the crop production systems were measured using traditional microbiological sampling and real-time PCR.

## 2 Methods

### 2.1 Disinfection testing

The efficacy of four candidate sanitizers (5% ProSan, ozonated water, 3% hydrogen peroxide, and 3% hydrogen peroxide in ozonated water) for use in spacecraft was tested using four bacterial species (including biofilm and spore-forming bacteria) found in ISS potable water, because it is used to irrigate bioregenerative food systems (Ichijo et al., 2022; Stepanov et al., 2016; Novikova et al., 2006). The concentrations of the sanitizers used and the exposure times were chosen from previous work at the Kennedy Space Center (KSC). The bacterial isolates used included biofilm-producing organisms (*Pseudomonas aeruginosa* strain ATCC 10145, *Burkholderia cepacia* strain ATCC 25416, and *Sphingomonas paucimobilis* strain ATCC 29837), along with *Bacillus pumilus* strain SAFR-032, an endospore former that is resistant to disinfection. Cultures of these organisms were grown on Trypticase Soy Agar (TSA) (Becton Dickinson, Franklin Lakes, NJ, USA) at 30°C for 48 h. Colonies on these plates were scraped and suspended in 5 mL of sterile physiological saline to prepare a bacterial suspension with an optical density (OD) of 0.1 at 540 nm. These suspensions were diluted 1:100 by adding 50 µL of each suspension to tubes containing 4.95 mL of either 5% ProSan, ozonated water, 3% hydrogen peroxide, or 3% hydrogen peroxide in ozonated water with sterile water as a negative control. Each tube was vortexed for 10 s and sampled at 15 s and 30 s, and 1 min, 2 min, 5 min, 10 min, 30 min, and 60 min with the sampling time initiated at the vortexing step. At each time interval, 0.5 mL of sample was added to 4.5 mL of Dey-Engley neutralizing broth (Becton Dickinson, Franklin Lakes, NJ, USA) and vortexed again. Samples were serially diluted 10-fold with sterilized physiological saline and plated in duplicate on TSA plates before being incubated at 30°C for 48 h, after which the plates were removed from the incubator and any colonies present were enumerated to downselect the appropriate sanitizer for use in candidate system cleaning. The criterion for selection was achieving a 4 log reduction in a 15-second exposure.

### 2.2 Heat testing

The cleaning efficiency of the four heat treatments applied for up to 1 h was tested using biofilm-forming and spore-forming bacterial species. The second set of cultures of *P. aeruginosa* and *B. pumilus* was prepared as described above. Fifty-microliter aliquots of each suspension were added to test tubes containing 4.95 mL of sterile distilled water prewarmed to the following temperatures: 35°C, 70°C, 90°C, and 90°C with 3% hydrogen peroxide. These tubes were sampled by adding 0.5 mL of each solution to tubes containing 4.5 mL of room temperature sterile phosphate-buffered saline (PBS) at 15 s, 2 min, 5 min, 10 min, and 60 min. These tubes were vortexed for 10 s, and plate counts were acquired using the same plating methods described above. The criterion for selection was achieving >2 log reduction for spore-forming bacteria (i.e., *B. pumilus*).

### 2.3 Plant growth systems

Five sequential 28-day cropping cycles: three grow-outs of Mizuna mustard (*Brassica rapa* var. niposinica), followed by the fourth grow-out of red Romaine lettuce (*Lactuca sativa* cv. ‘Outredgeous’), and a fifth grow-out of Cherry Belle radish (*Raphanus sativus* var. ‘Cherry Belle’) were conducted using the control NFT hydroponic system and two candidate soilless systems (PTNDS and OD). The crops were grown in individual carts inside a growth chamber under typical environmental conditions found in the ISS growing conditions (i.e., 300 µmol m^−2^ s^−1^–450 µmol m^−2^ s^−1^ lighting during a 16 h/8 h photoperiod, 3,000 ppm CO_2_, and 23°C). However, the relative humidity was controlled at 65% instead of the 40% found on the ISS. The ISS is maintained at 40% for crew comfort but, more importantly, to minimize the proliferation of fungal species; thus, this test represents the worst case because of the expected higher fungal controls. The crops were grown using half-strength Hoagland’s solution (1,200 µS/cm) with a 100% NO_3_ formulation, and both the solution electrical conductivity (EC) and pH drift were adjusted daily. The solution pH was adjusted to 5.8 using 0.1 M HNO_3_ or 0.1 M NaOH. These adjustments were not automated and were performed manually during daily checks.

Each plant growth system had 0.4 m^2^ of cultivation area. A hydroponic NFT (Crop King Inc., Lodi, OH, USA) was used as the reference WNDS, because it is the standard method for commercial food production. Compared to the PTNDS and OD soilless watering systems, NFT plants generally emerged first, produced healthy seedlings with longer roots, produced more leaves, and had larger leaf areas because hydroponic plants have increased leaf turgor, which drives fast leaf expansion (Monje et al., 2019). The PTNDS and OD plants had ~35%–50% of the fresh mass of the NFT plants; thus, their planting densities were nearly doubled in this study to produce similar amounts of salad crops in each system. The planting densities in the NFT, PTNDS, and OD systems were 25 plants/m^2^, 45 plants/m^2^, and 40 plants/m^2^, respectively. The seedlings were covered using germination domes (circular Petri dish covers) during seed imbibition until thinning on day seven after planting.

**Figure 1:**
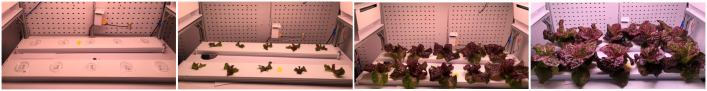
Outredgeous lettuce in two NFT troughs. Plants were seeded in wicks at a fixed planting spacing. The plants share a common root zone that is bathed by a thin film of recirculating nutrient solution.

The hydroponic NFT system had ten plants on 0.4 m^2^ arranged into two troughs, each with five plants (**Figure 1**). Seeds were planted at each position on Nitex wicks to keep the seeds wet through capillary wetting (Wheeler et al., 1996). The hydroponic roots formed thick mats inside the troughs and were bathed by recirculating the nutrient solution from a 15 L water reservoir flowing at ~1 L/min–1.5 L/min. After the shoots were harvested, the root mat in each trough was removed, wicks embedded in the root mat were removed, and the system was sanitized.

**Figure 2:**
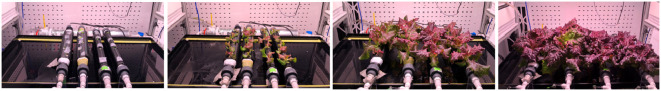
Outredgeous lettuce in four PTNDS tubes. The plants were seeded in porous tubes, and the planting spacing was varied as the plants grew. The seeds were planted in wicks over ceramic tubes and kept under suction. The tubes were wrapped with a 3D printed shell with a removable cover.

The PTNDS system had 18 plants planted in 0.4 m^2^ arranged into four ceramic porous tubes (**Figure 2**). The PTNDS consisted of two ‘double’ tubes and two ‘single-tube’ configurations irrigated from a 3 L reservoir. The double-tube configuration contains two tubes running side-by-side to increase the rooting surface area. It contained five plants, and the single tubes contained four plants. Seeds were planted at each position onto 5 mm thick, 1 cm polyurethane foam squares placed on the tubes. The tubes were wrapped in a 3D printed shell with a removable cover. Following harvest of the shoots, the 3D printed tube covers were removed. Dense root mats growing over the tubes were removed by cutting with a razor blade along each tube.

**Figure 3:**
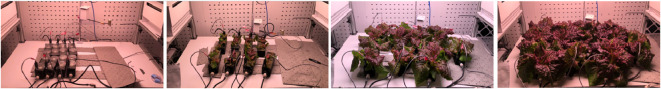
Outredgeous lettuce in eight soaker boxes. The plants were seeded, and the planting spacing was varied as the plants grew. The seeds were planted in foam held in the soaker box lids and the 3D printed soaker boxes were watered using an ebb and flow watering system.

The OD system had 16 plants (0.4 m^2^) arranged in eight rectangular soaker boxes with two plants per box (**Figure 3**). Seeds were planted at each position onto 5 mm thick, 10 cm^2^ polyurethane foam inserts held in the lids of the soaker boxes. Roots grew out of the foam into the soaker box, where they were watered from a 5 L reservoir using 17-minute flood/drain cycles. The soaker boxes were not drained entirely during the flood/drain cycle as the plants matured to have a buffer to prevent wilting in case of system failure. After the shoots were harvested, a thick rectangular root mat of white roots was removed from each soaker box.

### 2.4 Cropping cycles

Five consecutive 28-day crop cycles (i.e., three crops of Mizuna, followed by lettuce and radish crops) were conducted using the control NFT hydroponic system and two soilless systems (i.e., PTNDS and OD). The hydroponic control system was sanitized using a control sanitization protocol, and the two soilless systems were sanitized using a sanitization protocol developed for spacecraft. This approach was designed to mimic the continuous operation of a space crop production system, with a variety of crops exhibiting variable growth rates with prescribed sanitization events between each crop cycle. However, the crop productivity from these sequential harvests have not yet been reported. There were significant differences in cultivation (i.e., planting wick configurations, light levels, and moisture setpoints) during different phases of germination that were used to optimize seedling establishment in the PTNDS and OD systems. Although the cultivation configurations were not identical in all crop cycles, each WNDS had a full crop load that grew under the same environmental conditions and required sanitization and microbial monitoring after each harvest.

**Figure 4:**
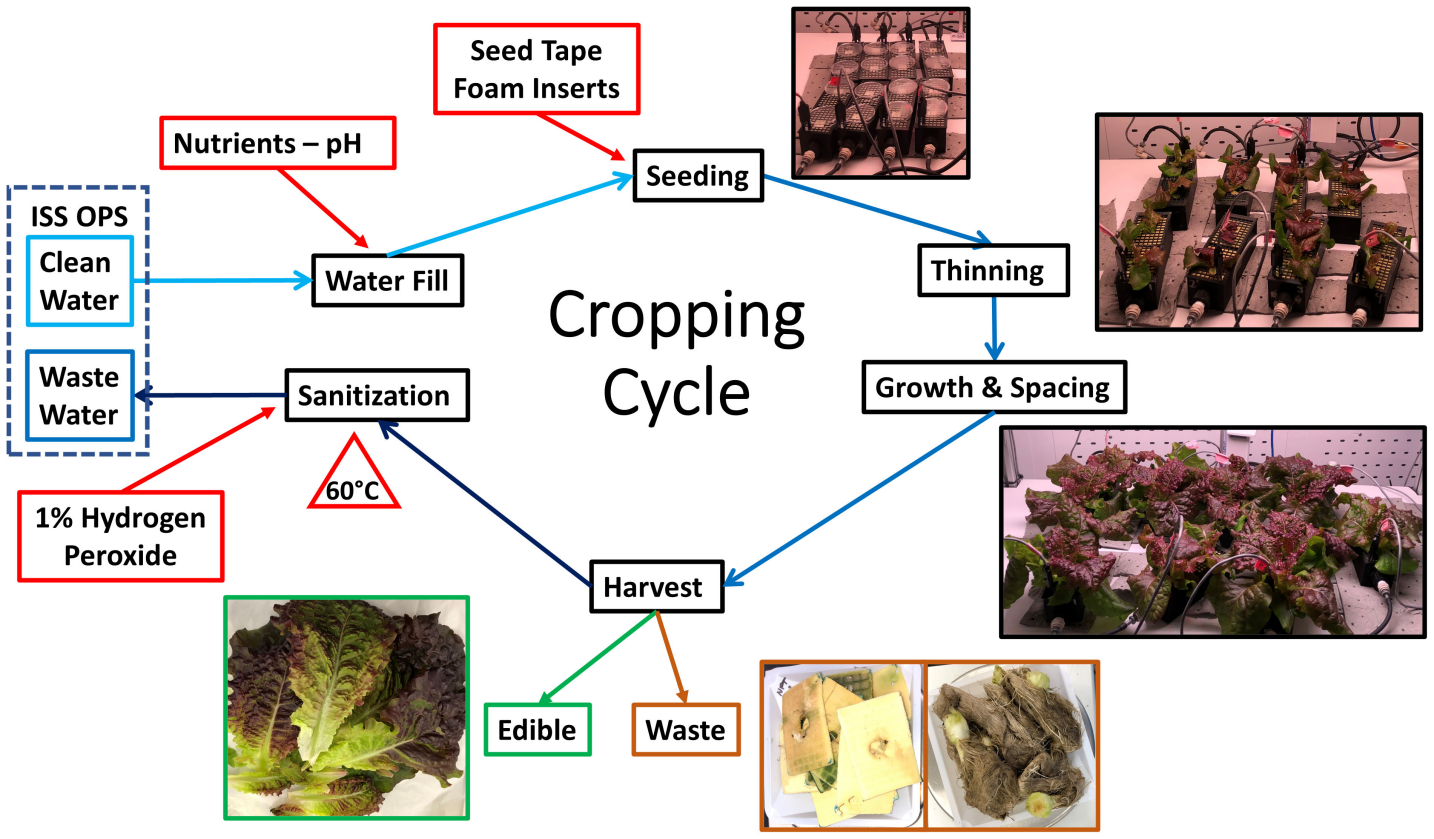
Soilless WNDS cropping cycle. The nutrient solution reservoir was filled with 5 L ½ Hoagland’s nutrient solution. WNDS were seeded, plants were thinned, plant spacing was changed as plants grew, plants were harvested, and wicks/foam/roots were discarded. The nutrient solution was discarded and 5 L of 1% hydrogen peroxide sanitizer was added. The system was heat sterilized at 60°C for 1 h. The sanitizer was recirculated for 12 h and discarded prior to starting the next crop cycle.

Each cropping cycle (**Figure 4**) included five steps.

#### 2.4.1 Water fill

The NFT, PTNDS, and OD reservoirs and water lines required for water delivery to the plants were filled with half-strength Hoagland’s solution. The NFT reservoir contained 15 L of a recirculating nutrient solution. The reservoirs of the PTNDS and OD systems held 3 L and 5 L of nutrient solution, respectively.

#### 2.4.2 Seeding and germination

The systems were planted with four seeds per location. Seeds were held in Nitex wicks in the NFT, preplanted polyurethane foam squares were placed on the porous tubes of the PTNDS, and preplanted foam was inserted into the OD soaker box lids. The seeds were flooded for imbibition, and a nutrient solution was provided in flood/drain cycles to both porous tubes and soaker boxes. Germination domes were used during the first three days to maintain high local humidity near the germinating seedlings.

#### 2.4.3 Thinning

Thinning occurred on day seven after planting. Only one of the four planted seeds was retained. The seedlings reflected the average size of each WNDS.

#### 2.4.4 Grow spacing

NFT had a fixed plant spacing between the plants. The PTNDS and OD systems employed a variable plant spacing scheme that increased the energy efficiency of crop production, as light capture was optimized. The spacing between the porous tubes and soaker boxes was adjusted manually at daily intervals as the plants grew to fill the entire growing area.

#### 2.4.5 Harvest

At 28 days after planting, the plants in each WNDS were harvested, generating edible biomass and waste consisting of roots, used foam, or wick inserts. The crop yield was determined by the edible fresh mass (e.g., lettuce, Mizuna leaves, or radish bulbs) and the inedible portion, which included the remaining stems and roots. The roots, used foam, or wicks determined the amount of waste generated per cropping cycle that was disposed from each WNDS.

#### 2.4.6 Sanitization protocols

The goal of the sanitization protocols was to maintain the crop production systems clean throughout sequential cropping cycles. The WNDS components were sanitized after each harvest and the sanitization protocols were consistent across all five crop cycles.

The hydroponic control system was sanitized using a control sanitization protocol that consisted of scrubbing the reservoir and trough surfaces with 3% bleach, rinsing, and soaking for 12 h in 3% hydrogen peroxide. It was developed at the KSC in the 1990s and is consistent with the commercial sanitization protocols. The PTNDS and OD soilless systems were sanitized using a protocol derived from the sanitizer screening and heat sterilization tests described above. The reservoirs were filled with 1% hydrogen peroxide, and the sanitizer was recirculated throughout the watering system for approximately 12 h using the same fill/drain cycles used to grow the plants. The 1% hydrogen peroxide sanitizer solution was heated to 60°C during the first hour to dislodge biofilms from the PTNDS and OD components (reservoirs, fittings, tubing, and root modules). Although the two screening tests suggested that a combination of heat (90°C for 1 h) and 3% hydrogen peroxide soaking would meet the selection criteria, the sanitization protocol adopted for this study was relaxed for two reasons: 1) producing a 1% hydrogen peroxide sanitizer onboard spacecraft is probably achievable in the near future, and 2) the 3D printed root modules used in the PTNDS and OD soilless crop production systems deformed at 70°C.

Once sanitized, the hydrogen peroxide solutions in the reservoirs were discarded into a wastewater reservoir prior to refilling each WNDS with fresh half-strength Hoagland’s solution to start a new crop cycle. The sanitization solution was removed from each WNDS because preliminary tests showed that 1% hydrogen peroxide inhibited seed germination in the next crop.

### 2.5 Microbiological analysis of plant growth systems

**Figure 5:**
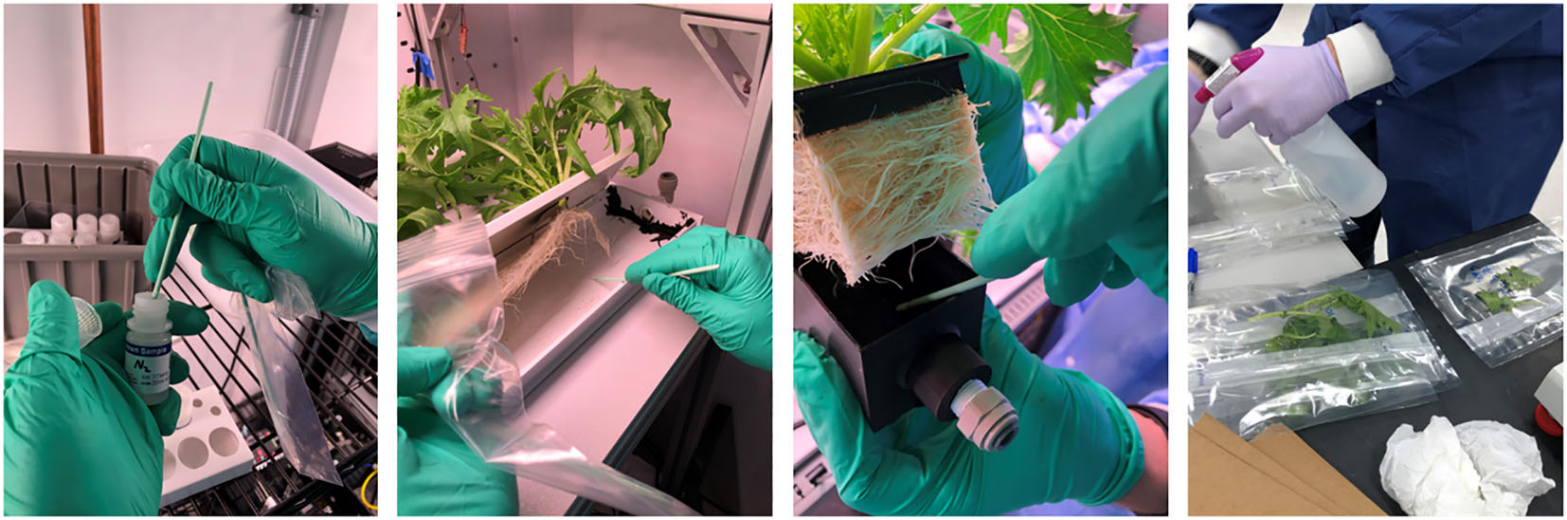
Collection of swab samples at harvest. The areas of the reservoirs (1 inch^2^), rooting surfaces from PTNDS porous tubes, NFT trays, and OD soaker boxes were swabbed from the selected sites and saved into vials. The edible biomass samples were collected in sterile bags. The swab and biomass samples were analyzed for APC and Y&M.

Microbial analysis conducted on water delivery systems identified that measuring the microbial and fungal counts from nutrient solution reservoirs, root module surfaces in contact with roots, and plant samples was needed to verify that the sanitization plan adopted was effective (Monje et al., 2019). Surface swabs (1 in^2^ areas) from randomly selected sites were collected following harvest and system sanitization events. Three surface samples were collected from the root modules (i.e., trough, porous tube, or soaker box) and another from the nutrient solution reservoir (i.e., a wall) for each nutrient delivery system (**Figure 5**). Cleanroom swabs (TX759B Swab, Texwipe, Kernersville, NC, USA) were placed in 10 mL PBS with 0.3% Tween and vortexed for 2 min to dislodge cells from the swab surface before utilizing the resultant fluid for further testing. The samples were analyzed for bacterial aerobic plate counts (APC) and fungal yeast and mold (Y&M) plate counts by diluting the vortexed swab solution (0.5 mL) in tubes containing 4.5 mL of sterile PBS. These samples were diluted 1:10 to appropriate concentrations before being plated onto TSA plates to obtain aerobic plate counts, and Inhibitory Mold Agar (IMA) plates for yeast and mold counts. The plates were then incubated at 30°C for 48 h and 120 h. Following incubation, the plates were removed, and colonies were enumerated.

At each harvest event, three randomly selected plants were collected from each watering system for microbial analysis of the fresh edible biomass. The samples were placed into mixing bags and blended using a bag mixer (BagMixer 400 W, Interscience, Saint Nom la Brétèche, France) for 2 min with 30 mL of sterile PBS. After mixing, the samples were serially diluted in PBS, and the three highest dilutions were plated in duplicates on TSA and IMA. The agar plates were incubated and enumerated using the parameters described above.

### 2.6 PCR processing with RAZOR EX

In this study, the RAZOR EX real-time polymerase chain reaction (PCR) System (BioFire Defense, Salt Lake City, UT, USA) was used as a potential inflight method to monitor the effectiveness of the sanitization protocols in spacecraft. RAZOR EX was successfully used to measure microbial counts from potable water samples on ISS and from swab samples of lab-grown tomatoes at KSC (Khodadad et al., 2021). Quantitative real-time polymerase chain reaction (qPCR) provides a relative abundance value for microbial presence and is an effective method for detecting and quantifying microorganisms in environmental samples. RAZOR EX was not able to estimate Y&M plate counts because protocols for processing fungal samples were not available. RAZOR EX utilized in this study was designed as a sample-to-answer instrument in which the sample was directly introduced without requiring nucleotide isolation methods. The introduced sample was mechanically disrupted and the primers were annealed to the exposed DNA, followed by optimized PCR thermocycling conditions. This allows for the amplification of all intact 16S ribosomal RNA (rRNA) genes in near real-time, within 45 min of sample acquisition.

#### 2.6.1 Surface sample processing

The swabs processed for APC counts during the microbiological processing were used for downstream semi-quantification using RAZOR EX and customized pouches containing all PCR reagents (BioFire Defense, LLC, Salt Lake City, UT, USA). To run the liquid samples, the sample vials were first vigorously vortexed to resuspend any cells that may have settled or re-adhered to the swab. Then a syringe containing ~200 µL–300 µL and fitted with a cannula was inserted into each well port and allowed to freely draw in the appropriate volume into the vacuum-sealed RAZOR EX pouch, which contained lyophilized reagents, including a 16S universal primer combination and labeled probe (**Table 1**).

**Table 1:** RAZOR EX PCR cycling protocol for 16S rRNA reactions and primer combinations with TaqMan Probe.

Gene	Forward Primer 5’–3’	Reverse Primer 5’–3’	TaqMan Probe 5’–3’	
*rRNA*	CGGTGAATACGTTCYCGG	GGWTACCTTGTTACGACTT	CTTGTACACACCGCCCGTC	
# of Cycles	Denaturation Temperature (°C)	Denaturation Time (s)	Annealing Temp (°C)	Annealing Time (s)
1	94	240	56	60
41	94	15	56	60

Two positive control standards and a negative control were included in each pouch run for reference and relative quantification, respectively (Khodadad et al., 2021). To compare RAZOR EX values to heterotrophic plate count data, lower and upper boundary estimates were created based on known 16S copy numbers within the *E. coli* control standards and an estimate of cells/ng. The lower and upper boundaries were averaged, dilution factors were considered, and relative quantification was calculated and compared to heterotrophic plate counts (**Supplementary Material**).

#### 2.6.2 Plant sample processing

At harvest, three random plants were sampled from each WNDS system. Plants were placed in a stomacher bag with calculated volume of buffered peptone water (BPW) (30 mL for leaves and 50 mL for radish bulbs). Stomacher bags were vortexed, and the solutions were injected into vacuum-sealed RAZOR EX 16S pouches with lyophilized reagents. The thermocycler conditions are listed in **Table 1**. Real-time PCR analysis was completed for each sample and quantified based on the threshold value of the positive standards and their known concentrations. The conversion of the RAZOR EX 16S copy number to APCs is described above and in the **Supplementary Material**.

Edible plant material from the Outredgeous lettuce and Mizuna leaves could not be processed using RAZOR EX. Chloroplasts and mitochondrial DNA were released because of the maceration of the plant material. A search of the target TaqMan Probe sequence on NCBI BLAST revealed alignment in both leafy greens tested. Therefore, the RAZOR EX total heterotrophic count assay utilized in this study was not applicable for the identification of bacterial concentrations within plant materials that contain chloroplast or mitochondrial DNA but was valid for radish bulbs.

#### 2.6.3 Validation against culture-based plate counts

The microbial abundances measured using RAZOR EX were validated against culture-based plating and enumeration methods. The relative microbial abundance measured by RAZOR EX can be higher than traditional APC counts due to several factors. First, culturable microbes account for approximately 10% of the total microbial community in some cases, as some microbes will grow at slower rates or not on the selected media used in this study and are therefore not detectable in the APC (Byrd et al., 1991; Kalmbach et al., 1997). Second, qPCR can amplify DNA fragments from dead cell tissue, if intact, allowing unique primers to anneal and then amplify (Taylor et al., 2014). Third, the 16S rRNA gene can amplify chloroplast DNA (from host cells, if present), thereby increasing microbial cell counts. Fourth, the 16S rRNA gene is present in numerous copies per cell and may even vary in the number of copies in cells of the same species, providing only the relative abundance of microbial cell counts. Although in many cases the constituents can be identified, the number of 16S rRNA genes varies between species, and in some cases, may vary in number between the cells of a single species (Ibal et al., 2019). For example, a study conducted by Pei et al. (2010) described over 425 bacteria with between two and 15 copies of the 16S rRNA gene. *B. pumilus* had seven copies of the rRNA gene, whereas *B. subtilis* had 10 copies. Various *Burkholderia* species have one to six copies. This variation makes it difficult to provide accurate abundance measures using this method, so we used relative abundance, which may be one to two logs higher than that measured by culture-based methods.

### 2.7 Statistical analysis

Data from microbiological plate counts were log-transformed and compared using one-way ANOVA followed by Tukey’s multiple comparisons test using the NFT samples as the control group. Additional comparisons were performed with paired t-tests of pre- and post-sanitization swab samples from both the reservoirs and root module surfaces. This analysis was performed using GraphPad Prism version 9.0.0 for Windows (GraphPad Software, San Diego, CA, United States).

## 3 Results and discussion

Bioregenerative crop production systems must be sanitized after each crop cycle to minimize food safety hazards and biofilm formation within watering systems. The cleaning efficiency of the sanitization method was determined by measuring the microbial and fungal loads from the nutrient solution reservoirs, root module surfaces in contact with roots, and edible plant samples (leaves and radish bulbs) grown in each watering system. Two methods were used to measure the system microbial load obtained from the swab samples. The first was microbiological sampling, which measured APC and Y&M counts. The APC method, the traditional laboratory standard that measures the number of heterotrophic aerobic culturable colonies per unit, cannot be implemented in spacecraft because it requires extensive laboratory capabilities. Thus, the food safety of lettuce plants grown in Veggie on ISS has been assessed using mature leaf samples returned to Earth for analysis (Khodadad et al., 2020; Hummerick et al., 2021; Bunchek et al., 2024). A second method was used in this study to compare the sanitization results with conventional plate counts. Microbial swabs were analyzed using RAZOR EX, a portable PCR unit that quantifies total bacteria based on 16S rRNA. The RAZOR EX data can be readily measured in spacecraft in near real-time to determine the system cleaning efficiency.

### 3.1 Sanitizer screening

Sanitizing crop production systems minimizes contamination by potential human and plant pathogens and prevents the accumulation of biofilms. Hydrogen peroxide and aqueous ozone are used to sanitize and disinfect components of commercial plant growth systems. These solutions are strong antimicrobial agents and were selected for use because they eventually degrade into water and oxygen (Megahed, 2018; Mogren et al., 2018). Hydrogen peroxide is an effective biocide at concentrations as low as 1% for cleaning surfaces and has been demonstrated to be effective in cleaning *E. coli*-contaminated produce (Sapers and Sites, 2003). It can also be generated *in situ* in spacecraft, which could simplify the logistics of supplying hydrogen peroxide for sanitization in space (Vijapur et al., 2022). ProSan, a citric acid-based sanitizer that is generally regarded as safe for cleaning food preparation surfaces and as a produce wash, was included as a positive control. A formulation designed for use with produce wipes is currently being used on the ISS to clean Veggie-grown produce (Massa et al., 2017). ProSan wipes are also used to sanitize APH after each grow-out, as they are more effective than ethanol wipes and safe for use on food production surfaces. Aqueous ozone, a strong oxidizer, was also considered as a candidate sanitization agent because it is not corrosive, does not harm the skin, and can be generated *in situ* (Runia, 1994).

The efficacy of four cleaning solutions, 3% hydrogen peroxide, aqueous ozone solution, 3% hydrogen peroxide in an aqueous ozone solution, and 5% ProSan, was tested by adding bacterial suspensions to test tubes containing the desired cleaning solutions. Log reductions were measured at different exposure times (**Table 2**). In 15 s, 3% hydrogen peroxide (H_2_O_2_) reduced the numbers of *P. aeruginosa*, *S. paucimobilis*, and *B. cepacia* at least 4 logs. The 5% ProSan positive control, exhibited similar efficacy. Disinfectants were not very effective against *B. pumilus* spores, with 3% hydrogen peroxide being the most effective with a 2.18 log reduction after 1 h of exposure. Aqueous ozone alone was effective after 10 m of exposure but was not effective against *B. pumilus* spores. The peroxide/aqueous ozone mixture only marginally increased cleaning efficacy.

**Table 2:** Log reduction of organisms tested with chemical disinfection for down-selection.

Treatments	*Time*	*P. aeruginosa* (6.00)	*S. paucimobilis* (6.43)	*B. cepacia* (5.57)	*B. pumilus* (6.29)
Untreated control	–	0.07	−0.43	−0.43	−0.17
Ozone	15 s	0.16	−0.44	0.40	–
	10 min	4.29	1.58	2.43	–
	60 min	3.70	–	–	−0.20
Ozone with 3% H_2_O_2_	15 s	3.82	1.28	4.29	–
	10 min	4.00	3.82	4.00	–
	60 min	4.29	–	–	1.09
3% H_2_O_2_	15 s	4.29	4.29	4.00	–
	10 min	3.82	4.29	4.00	–
	60 min	4.00	–	–	2.18
5% ProSan	15s	4.00	4.00	3.82	–
	10 min	3.70	4.00	4.29	–
	60 min	3.82	–	–	−0.30

Starting Log_10_ values are shown in parenthesis. Dashes indicate testing parameters not performed for a given organism.

### 3.2 Heat sterilization testing

Commercial sanitization often uses boiling water at 100°C to kill pathogenic bacteria that cause waterborne diseases (Runia et al., 1988). Hot water is non-toxic and can be generated *in situ*. Preliminary testing demonstrated that hot water (70°C) quickly removed the attached biofilms from the porous tubes and tubing. This makes heat sterilization a suitable replacement for methods that employ hardware disassembly, followed by manual scrubbing. Heating solutions are easier to accomplish in spacecraft, reduce crew time, and eliminate the handling of cleaning agents with high chemical toxicity levels.

The efficacy of heat sterilization at 35°C, 70°C, 90°C and 90°C with 3% hydrogen peroxide was also tested. The second set of tests included heating to 90°C in combination with either 3% hydrogen peroxide or sterile water to test the efficacy of heating as the sole source of sterilization. Heat sterilization at 35°C performed similarly to the 30°C control, with minimal changes during the period of exposure. Higher temperatures resulted in a marked increase in the reduction of culturable bacterial load at 70°C achieving a 4.3 log reduction after 15 s of exposure, and at 90°C achieving a similar 4 log reduction after 15 s of exposure. Heat sterilization proved ineffective as a cleaning method for spore-forming bacteria, with a minimal reduction noted for all samples except the 70°C sample after 1 h of exposure (**Table 3**). Because planktonic cells were effectively eliminated by heat exposure alone, the hydrogen peroxide and heat combination was exclusively tested on the *B. pumilus* spore culture at 90°C with 2.8 and 3.4 log reductions noted after 1 h and 2 h of exposure, respectively (**Table 3**).

**Table 3:** Log reduction of organisms tested with heat sterilization.

1 Treatments	Time	*P. aeruginosa* (6.00)	*B. pumilus* (6.00)
2 Untreated control		−0.08	0.40
35°C	15 s	0.11	–
1	2 min	1.00	–
1	5 min	0.35	0.16
1	10 min	–	0.04
1	60 min	–	0.14
70°C	15 s	4.29	–
1	2 min	4.29	–
1	5 min	4.00	0.12
1	10 min	4.00	0.03
1	60 min	–	2.01
90°C	15 s	4.00	–
1	2 min	4.00	–
1	5 min	3.82	−0.06
1	10 min	3.82	−0.05
1	60 min	–	0.13
90°C + 3% H_2_O_2_	60 min	–	2.77
1	120 min	–	3.37

Starting Log_10_ values are shown in parenthesis. Dashes indicate testing parameters not performed with a given organism.

Despite its success in reducing the microbial load of planktonic cells, the 3% peroxide solution alone was ineffective when applied to *B. pumilus* spores (achieving only a 2.2 log reduction after 1 h of exposure, **Table 2**). Similarly, the application of heat alone was also ineffective in this removal; however, when 3% hydrogen peroxide and 90°C heat were applied simultaneously, the number of viable spores was significantly reduced, demonstrating the need for multi-faceted cleaning and sanitization approaches. Frequent application of cleaning methods is recommended to avoid the biofilm formation as they have been shown to be more resilient when presented with hydrogen peroxide as a sanitizer (DeQueiroz and Day, 2007).

### 3.3 System sanitization procedure

Routinely producing or mixing several liters of 3% hydrogen peroxide in a spacecraft is not trivial. On Earth, this was accomplished by dispensing sufficient 30% hydrogen peroxide to the water in the solution reservoir to reach the 3% concentration needed for sanitization. In space, a high-THL sanitizer (e.g., bleach or 30% hydrogen peroxide) would have to be handled (i.e., dispensing concentrate to prepare a dilute solution) in a glove box and entail much more crew time than handling 1%–3% hydrogen peroxide produced electrochemically *in situ* (Vijapur et al., 2022).

Based on the results of the sanitizer screening tests, the most effective sanitization protocol for controlling both biofilms and spore-forming bacteria was the combination of 3% hydrogen peroxide sanitization and heat sterilization at 90°C for 1 h. However, the sanitization protocol adopted in this study sought to minimize risks to the crew associated with handling sanitizers in spacecraft, as well as to accommodate the thermal compatibility considerations of the 3D printed materials used. Therefore, the optimal sanitizer concentration and heat sterilization temperature were reduced, and the PTNDS and OD systems were sanitized by heat sterilization at 60°C for 1 h followed by a 12-hour soaking in 1% hydrogen peroxide. These compromises probably indicate that the sanitization protocol used in this study was not effective for removing spore-forming bacteria.

The heat sterilization temperature was lowered from 90°C to 60°C due to thermal limitations of the PTNDS and OD systems. During the pre-planting cleaning cycle, it was discovered that the 3D printed polycarbonate used to construct the custom-made PTNDS tube connectors and OD soaker boxes was structurally compromised (i.e., they would become deformed) when exposed to 70°C or warmer solutions required for heat sterilization.

It is feasible to implement the proposed cleaning protocol in a spacecraft environment. The sanitizer (2%–3% hydrogen peroxide produced *in situ*) is first introduced into the space crop production reservoir and diluted to the desired concentration (1% hydrogen peroxide) using potable water. Water can then be heated using an inline heater. A 600 W heater can heat a 5-liter reservoir to 90°C in 52 min and 60°C in 35 min (see **Supplementary Material**).

### 3.4 Heterotrophic plate count analysis of sequential crop cycle swabs

The efficacy of the sanitization protocol was evaluated over five sequential crop cycles. Swab and plant samples were collected before harvest and after the post-harvest sanitization period was completed. The sampling sites (one nutrient solution reservoir and three root module locations from each watering system) were chosen randomly, but the same locations were swabbed during pre- and post-harvest sampling. The microbial loads from the surface swabs in each WNDS were measured after harvest and after sanitization. Microbial and fungal loads from edible biomass were measured after harvest for each WNDS.

Although no microbial food safety standards have been specified by NASA for the consumption of space-grown produce, microbiological standards for non-thermostabilized foods can be used as a guideline for acceptable levels of bacteria and fungi. The NASA safety standards for non-thermostabilized foods are a) APC—2 × 10^4^ CFU/g (4.3 log_10_ CFU/g) fresh weight of a single sample or 1 × 10^4^ CFU/g (4.0 log_10_ CFU/g) fresh weight for two samples, and b) Yeast and Mold—1 × 10^3^ CFU/g (3 log_10_ CFU/g) fresh weight of a single sample or 1 × 10^2^ CFU/g (2.0 log_10_ CFU/g) for two samples (Perchonok et al., 2012). In addition, microbial and fungal loads of produce should be compared to those found in fresh market produce and lettuce grown on ISS. For market produce, the mean APC for lettuce, celery, cauliflower, and broccoli were 8.6 log_10_ CFU/g, 7.5 log_10_ CFU/g, 7.4 log_10_ CFU/g, and 6.3 log_10_ CFU/g, respectively. The mean Y&M counts for lettuce, celery, cauliflower, and broccoli were 5.0 log_10_ CFU/g, 4.9 log_10_ CFU/g, 4.8 log_10_ CFU/g, and 4.6 log_10_ CFU/g, respectively (Thunberg et al., 2002). In contrast, microbial and fungal counts of lettuce from Veggie on ISS ranged from APC 2.1 log_10_ CFU/g–4.8 log_10_ CFU/g and Y&M 2.3 log_10_ CFU/g–4.3 log_10_ CFU/g, respectively (Khodadad et al., 2020).

#### 3.4.1 System reservoirs

**Figure 6:**
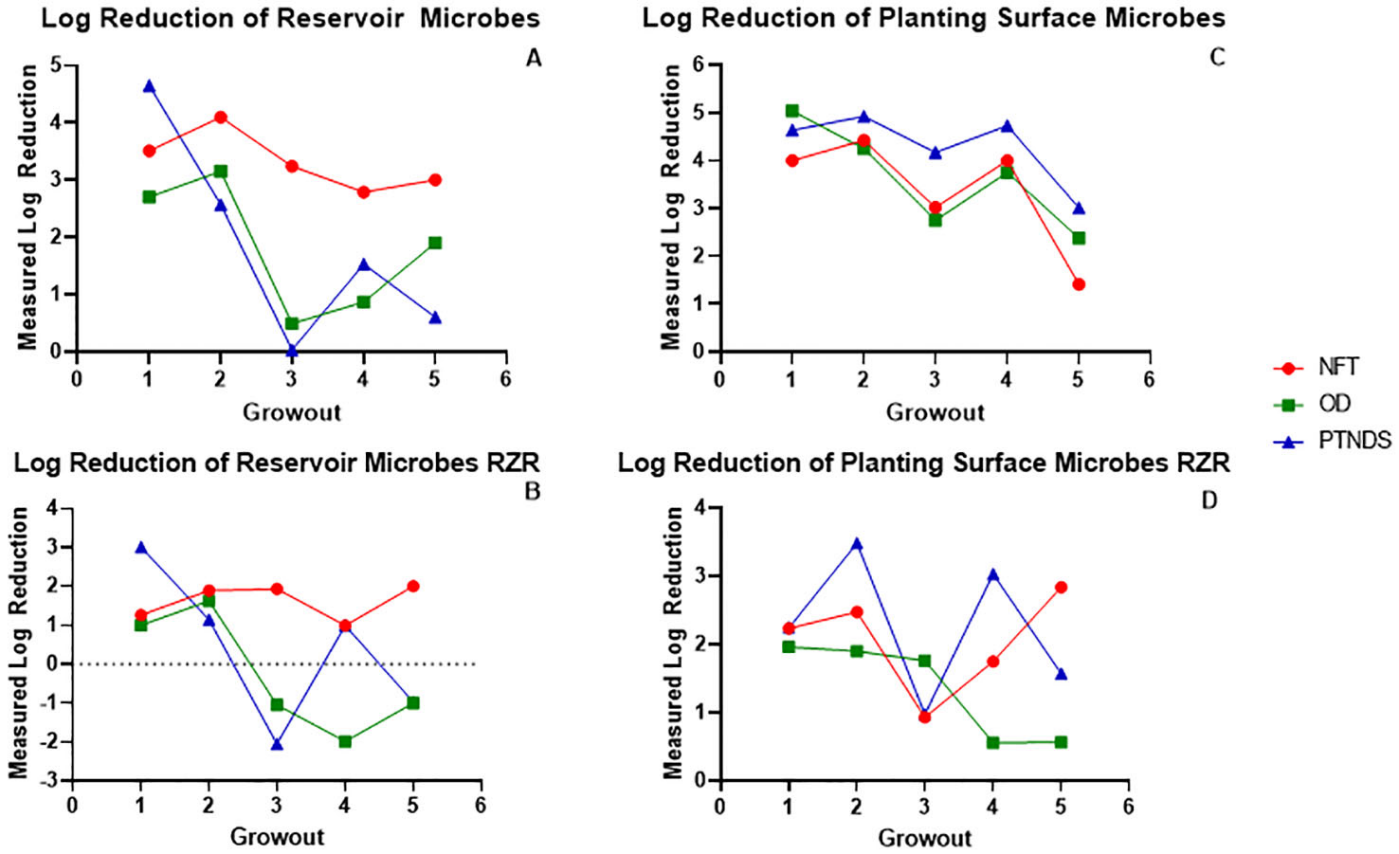
Log reductions observed in reservoirs and root modules. The log reductions after sanitization measured with APC and RAZOR EX were plotted as a function of the grow-out. Grow-outs 1–3 were Mizuna mustard, grow-out 4 was ‘Outredgeous’ red romaine lettuce, and grow-out 5 was Cherry Belle radish. **(A)** Reservoir APC data, and **(B)** RAZOR EX data show reduced cleaning efficiency in PTNDS and OD after the second grow-out compared with NFT. **(C)** Root module APC data show that the surfaces of the PTNDS and OD WNDS remain as clean as NFT after five grow-outs. **(D)** Generally, RAZOR EX data show lower and more variable log reductions than APC data.

The log reductions in microbial counts after sanitization (post-harvest counts–post-sanitization counts) for reservoirs and root surfaces during the five sequential crop cycles are shown in **Figure 6**. The APC log reductions in the NFT system reservoir sanitized with 3% bleach followed by 3% H_2_O_2_ remained within 3–4 during the five sequential grow-outs (**Figure 6A**, red). However, the APC counts of the PTNDS (blue) and OD (green) reservoirs, sanitized with heated 1% H_2_O_2_ only, remained nearly as clean as the NFT for only two grow-outs and fell to <2 log reduction after the second grow out. Thus, the simpler PTNDS and OD cleaning procedures were effective for the two grow-outs, but then their efficacy dropped to approximately a 1–2 log-reduction. The post-sanitization log reductions for the reservoirs were also measured using the RAZOR EX (**Figure 6B**). The RAZOR EX data mirror the APC results, where the NFT cleaning protocol maintains a 1–2 log reduction throughout, and the PTNDS and OD systems are clean during the first two cycles. However, the RAZOR EX data showed negative log reductions for the PTNDS and OD system reservoirs, as the efficacy of the simpler PTNDS and OD cleaning procedures failed to clean the reservoirs. In contrast, APC log reductions on root surfaces from the PTNDS and OD systems were as clean as the NFT during all five grow-outs (**Figure 6C**). There was a ~2 drop in APC log reductions during the fifth crop cycle for all three WNDS systems, as radish crops had higher microbial loads on their root surfaces. The RAZOR EX data gathered from the root surfaces demonstrated a similar pattern to that of the measured reservoir log reduction using the same technique, in that there was a general decrease in the efficacy of the simpler cleaning method utilized for the PTNDS and OD systems by the end of the final grow-out (**Figure 6D**). The OD log reduction was maintained at ~2 for all Mizuna grow-outs, with diminished efficacy for grow-outs 4 and 5. The measured log reduction in the PTNDS was more variable, ranging from 1 to 3.5, averaging ~2.5, across all five grow-outs. The evidence of increased efficacy using a multi-step cleaning approach was again demonstrated in the NFT data, with an increase in efficacy being seen between grow-outs 3 and 4, as well as 4 and 5. Generally, the RAZOR EX data from the root module surfaces are highly variable and more difficult to interpret than the corresponding APC data.

**Figure 7:**
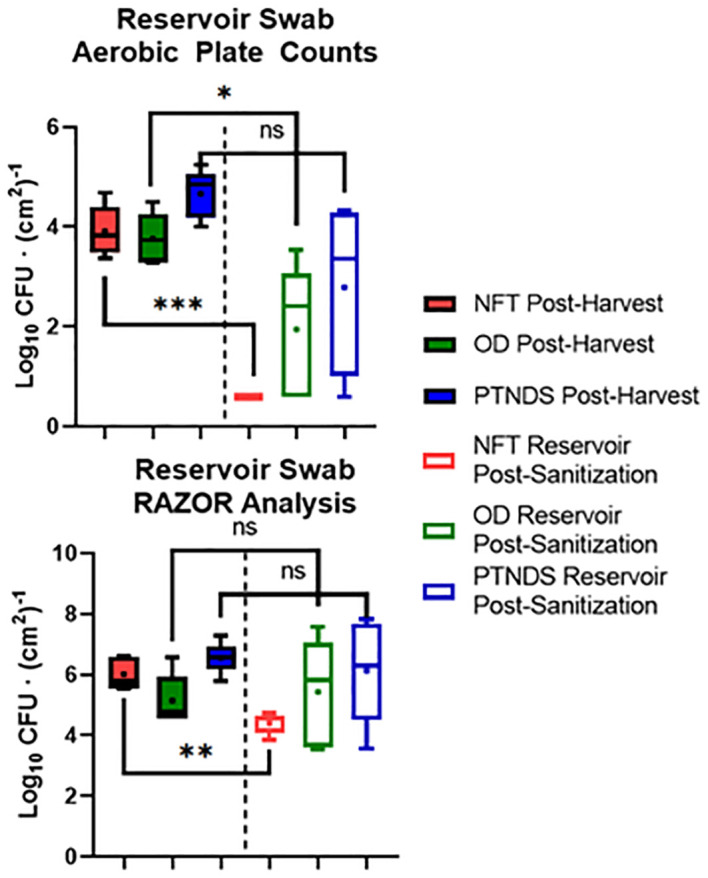
Aerobic plate count (top) and RAZOR analysis (bottom) of water system reservoir swabs. Randomly selected locations within the nutrient solution reservoirs of each plant growth system were swabbed following harvest (solid) and sanitization (empty) events during each crop growth cycle (n = 5). Error bars represent the minimum and maximum values, respectively. Asterisks (*) indicate statistical significance of the difference found between post-harvest and post-sanitization samples: ns, not significant, * = p <0.05, ** = p <0.005, *** = p <0.0005. Significant differences were determined using ANOVA with Tukey’s posttest to compare groups.

**Figure 7** (top) shows the average nutrient solution reservoir APCs from five sequential (i.e., three Mizuna, one lettuce, and one radish) cropping cycles for each WNDS. The post-harvest APC for the NFT, OD and PTNDS reservoirs ranged from 3.7 log CFU (cm^2^)^−1^ to 4.6 log CFU (cm^2^)^−1^. The NFT control system reservoir was consistently below the detection limit following system cleaning. The OD system reservoir showed a significant 2 log reduction, whereas the PTNDS system reservoir was not significantly cleaner. RAZOR EX analysis of the post-harvest swabs from the reservoirs (**Figure 7**, bottom) indicated approximate cell counts of 10^6^, 10^5^, and 10^6^ for NFT, OD, and PTNDS, respectively. The RAZOR EX post-harvest counts were approximately 2 logs higher than the measured APCs. Only NFT reservoir was significantly different between harvest and cleaning. The post-sanitization qPCR relative quantification indicated 10^4^ cells for the NFT and was significantly reduced (P <0.05), while both the OD and PTNDS reservoir cell counts were approximately 10^6^, indicating no significant changes after sanitization.

qPCR analysis of cell recovery from swabbed regions of the reservoir tanks indicated that only the NFT cleaning protocol was effective. This aligns with the APC count data; however, the quantitative count values were higher with qPCR. The RAZOR data for the NFT, PTNDS, and OD systems revealed a 3-log fold difference between the two detection methods. This may be explained by qPCR quantifying microbial populations through DNA amplification, rather than by organism proliferation. Therefore, it can detect and enumerate organisms that are not culturable by using standard methods. Additionally, it is not limited to the detection of living organisms and amplifies the DNA of dead cells and, in some cases, can slightly inflate the measured microbial abundance due to the presence of multiple rRNA genes in some organisms.

The log reduction data from both microbial monitoring methods (APC and RAZOR EX) confirmed that there were significant differences between NFT and the less stringent PTNDS and OD cleaning protocols. The microbial monitoring methods coincided in demonstrating that the PTNDS and OD protocols were effective only during the first two crop cycles (**Figure 6**), whereas the more stringent NFT protocol kept the reservoirs clean during the five sequential crop cycles (**Figures 6**, **7**).

#### 3.4.2 Plant root modules

**Figure 8:**
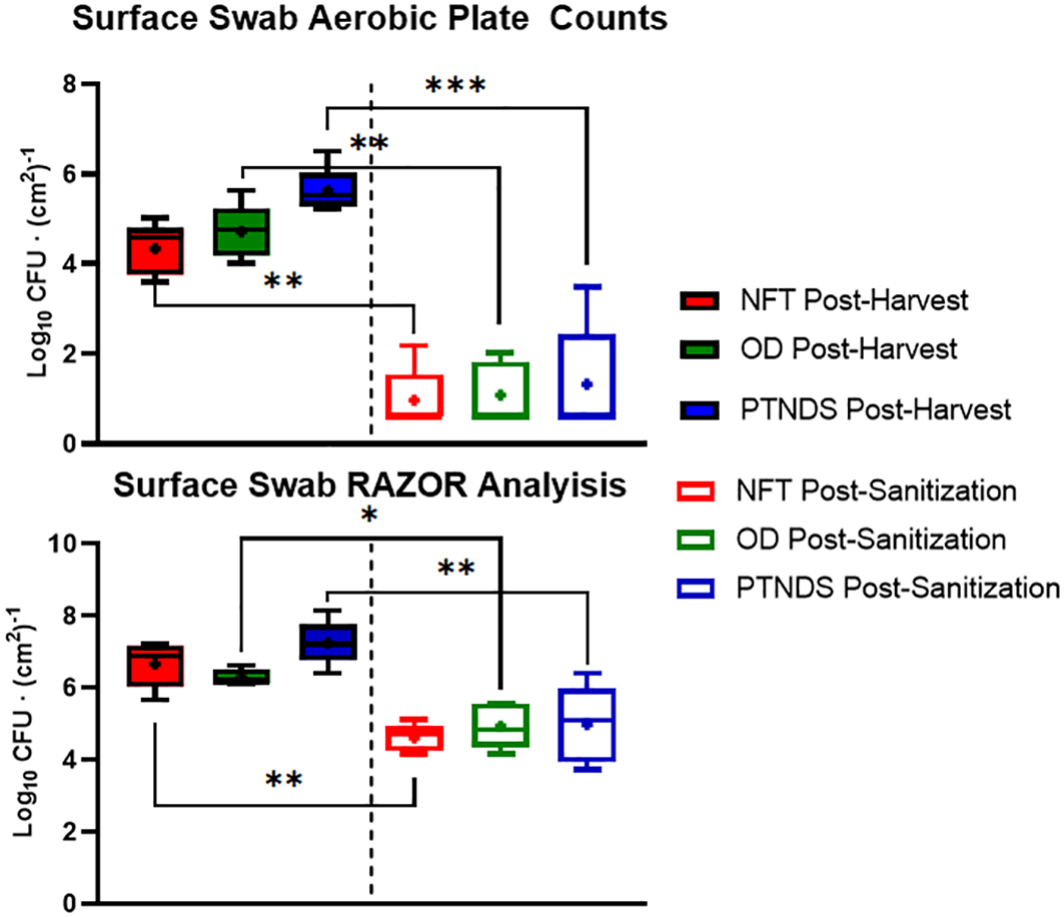
Aerobic plate count (top) and RAZOR analysis (bottom) of root module surface swabs. Randomly selected locations of each plant growth system were swabbed following harvest (solid) and sanitization (empty) events during each crop growth cycle (n = 5). Error bars represent the minimum and maximum values obtained. Asterisks (*) indicate statistical significance of the difference found between post-harvest and post-sanitization samples: * = p <0.05, ** = p <0.005, *** = p <0.0005. Significant differences were determined using an ANOVA with Tukey’s posttest to compare groups.

**Figure 8** (top) shows that swabs taken from the root module surfaces that were in contact with plant roots showed greater sanitizer efficacy than the reservoir swabs. Across all crop cycles, these crop contact surfaces were at or above the NASA safety standard level for non-thermostabilized foods (3.0 log_10_ CFU/g) prior cleaning and sanitization. Following system sanitization, the APCs of all WNDS systems were below this standard. qPCR analysis of the pre- and post-sanitization surface swabs also indicated that there was a significant reduction in the microbial load after cleaning of systems (**Figure 8**, bottom). Both measures, obtained with APCs and qPCR, showed 1–2 log reductions after cleaning, but the qPCR loads were orders of magnitude higher than the aerobic plate counts. The APC results suggest that the surface of the tubes in the PTNDS system has higher microbial loads (almost 10^2^ time higher) than those in the NFT and OD systems.

Data from the PTNDS cleanliness test from both microbial monitoring methods confirmed that there was a significant difference in microbial loads pre- and post-sanitization. This difference was detected even when there was a relatively small difference between the pre- and post-sanitization values measured via RAZOR when compared to the large difference observed in the APC measurements for the same samples. The APCs indicated that the sanitization procedure was effective; however, the RAZOR log reductions were smaller because it detected DNA from dead bacteria that remained in the root modules.

#### 3.4.3 Edible biomass APC

**Figure 9:**
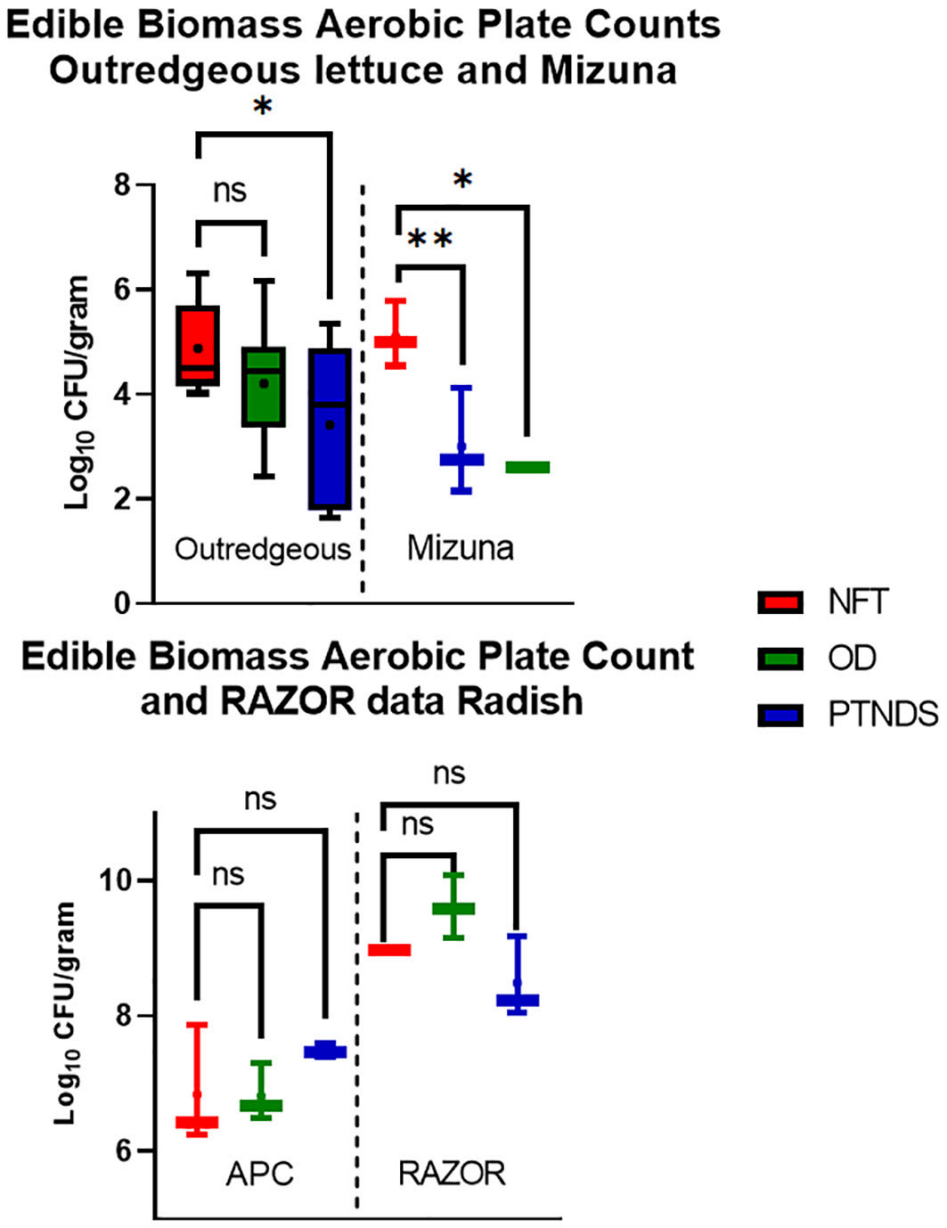
Aerobic plate count (top and bottom left) and applicable RAZOR analysis (bottom right) of edible biomass. Three plants per crop cycle were selected from each plant growth nutrient delivery system for the analysis. In contrast to APC, only RAZOR qPCR of non-photosynthetic radish bulbs was possible. Error bars represent the minimum and maximum values obtained. Asterisks (*) indicate statistical significance of the difference found between post-harvest and post-sanitization samples: ns, not significant, * = p <0.05, ** = p <0.005. Significant differences were determined using an ANOVA with Tukey’s posttest to compare groups.

The APCs of edible biomass in the soilless OD and PTNDS systems were compared with those of NFT (**Figure 9**, top). Lettuce and Mizuna plants grown in NFT exceeded the standards for non-thermostabilized foods (i.e., 4.9 vs. the 4.0 log−_10_ CFU/g standard), but they remained below the microbial loads of fresh market produce. The APCs of OD lettuce were lower than those of the NFT-grown lettuce and had an average log value of 4.20. Lettuce grown using the PTNDS system had a significantly lower culturable bacterial load (p <0.05) than NFT-grown lettuce, with a mean value of 3.4, and was below the current NASA safety standard for APC. Mizuna grown in both the OD and PTNDS systems had mean APC values of 2.60 and 3.00, respectively, which were significantly lower than those of the NFT (APC of 5.1) and below the safety standards for consumption. APC data from radish bulbs (**Figure 9**, bottom, left) harvested from all systems were well above the NASA safety standard for APC ranging from 6.81 to 7.48, but had similar microbial loads to fresh market produce.

The RAZOR EX RT-PCR system was also tested on processed plant material. Although the RAZOR qPCR analysis with the rRNA gene was accurate and sufficient with the surface and reservoir swabs, it was determined that the selected TaqMan primer and probe combination also amplified chloroplast DNA. Chloroplasts from both Mizuna and green lettuce over-inflated the relative abundance, and further investigation revealed a 100% match between lettuce and the16S rRNA gene primers of numerous combinations. The inability to remove the host DNA indicates that this method of detection is inappropriate for estimating the microbial abundance of fresh leaves. However, qPCR-based microbial abundance in non-photosynthetic plant tissues, such as roots or radish bulbs, can be quantified and analyzed. The radish bulbs did not contain chloroplasts; therefore, RAZOR EX was able to convert 16S read counts into accurate cell counts (**Figure 9**, bottom, right). The radish RAZOR 16S counts were approximately 2 log higher than those measured with APCs.

#### 3.4.4 Yeast and mold counts

**Figure 10:**
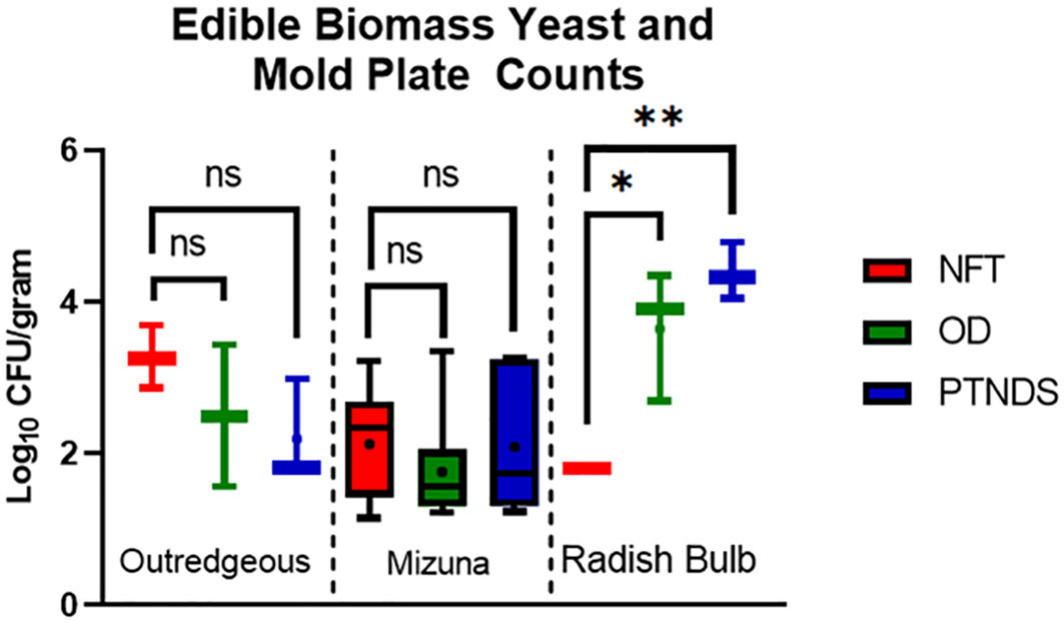
Yeast and mold plate count analysis of edible biomass. Three plants per crop cycle were selected from each plant growth nutrient delivery system for analysis by plating in duplicate on inhibitory mold agar (Mizuna n = 9; Outredgeous lettuce n = 3; Radish n= 3). Error bars represent the minimum and maximum values obtained. Asterisks (*) indicate statistical significance of the difference found between post-harvest and post-sanitization samples: ns, not significant, * = p <0.05, ** = p <0.005. Significant differences were determined using an ANOVA with Tukey’s posttest to compare groups.

Swabs for both reservoirs and root surfaces also yielded data on the IMA plates. However, nearly all pre-sanitization samples were overrun by a fast-growing white fungus, rendering the plates unreadable. However, after sanitization, all samples were below the detection limit (data not shown), which also brings the sampled root surfaces in line with being below the NASA safety standard for non-thermostabilized foods from the perspective of yeast, mold, and fungi. **Figure 10** shows that the lettuce and Mizuna plants grown in the NFT, OD, and PTNDS systems were below the NASA safety standard for non-thermostabilized foods of 3.0 log_10_ CFU/g threshold for yeast and mold. Radishes harvested from PTNDS, and OD were found to be above this benchmark and at least 2 log higher than those harvested from NFT with statistical significance indicated. Although these results suggest that radishes may be too contaminated to eat, they can be wiped with ProSan wipes for consumption, as this additional post-harvest cleaning step adds a ~2 log reduction in bacterial and fungal counts (Massa et al., 2017).

### 3.5 Differences between APCs and RAZOR-derived APCs

In some, but not all analyses, qPCR using the 16S rRNA gene indicated 1–2 log reductions in total microbial numbers in response to sanitization, confirming the plate count data (**Figures 7**, **8**), but the qPCR microbial counts were generally greater than the APC by two orders of magnitude. This may be explained by the fact that APC measures the live culturable microorganisms present in the sample, which is estimated to be approximately 10% of the microbes present. RAZOR EX detected the 16S rRNA gene from both living and non-living microbes present in the sample, as well as the number of copies of rRNA genes per cell. These differences had the potential to inflate the microbial counts, but the combination of the two methods provided a more accurate relative abundance of cells present in each sample. An alternative to qPCR identification and relative qualification would be whole-metagenome sequencing, which would provide broader identification of organisms and relative quantification. This is currently under investigation for the ISS (Lee et al., 2023).

## 4 Conclusion

A postharvest sanitization protocol for enabling sustainable space crop production was developed, and its efficacy was demonstrated during five sequential cropping cycles using microbial monitoring (**Figure 4**). The soilless PTNDS and OD WNDS were cleaned using the postharvest sanitization protocol, and the hydroponic NFT system was cleaned using a control sanitization protocol after growing Mizuna, lettuce, and radish crops. Two microbial methods (APC and Y&M plate counts) and a qPCR-based method (using a portable RAZOR EX unit) were used to measure microbial counts from swab samples taken from reservoirs, root module surfaces, and edible plant organs (leaves and radishes). In this study, qPCR microbial counts obtained with RAZOR EX were compared with APC microbial counts to evaluate whether qPCR methods (or improved systems such as miniPCR and MinION) can be used for near-real-time microbial monitoring in spacecraft.

Generally, the APC data indicated that the control sanitization protocol kept the NFT reservoir clean (i.e., ~3 log reduction) during the five grow-outs, but the post-harvest sanitization protocol did not adequately sanitize the PTNDS and OD reservoirs beyond the first two sequential crop cycles (**Figure 6A**). However, the soilless root modules were as clean as the NFT troughs during the five sequential cropping cycles (**Figure 8**), resulting in safe-to-eat plants (**Figure 9**). The Y&M data showed that the sanitization protocol was also effective for controlling yeast and mold in Mizuna and lettuce, but not in radish edible biomass (**Figure 10**).

The post-harvest sanitization protocol adopted (12-hour soaking in 1% hydrogen peroxide with 1 h initial heat sterilization at 60°C) was a compromise with respect to what sanitizer may be deployed in spacecraft and the thermal stability of the 3D printed components of the soilless WNDS used in this study. Thus, this may have resulted in fungal growth and biofilm formation in the reservoirs and in the lines transferring nutrient solution to the porous tubes and soaker boxes where the plants were grown. In spacecraft systems, however, WNDS subsystems are constructed with materials that can be heat-sterilized to 90°C, and improved sanitization of the PTNDS and OD reservoirs is expected. Nevertheless, this sanitization scheme produced clean produce for crew consumption over five sequential cropping cycles.

The qPCR data confirmed that the NFT reservoirs remained clean during the five sequential cropping cycles whereas the PTNDS and OD reservoirs remained clean for only the first two grow-outs (**Figure 6B**). These results indicate that qPCR data can be used for inflight microbial monitoring of crop production systems. The qPCR-based counts from reservoirs (**Figures 6**, **7**) and root module surfaces (**Figure 8**) generally corroborated the results from APC counts but were higher than plate counts. The RAZOR EX measures culturable and non-culturable bacteria but can also amplify DNA from dead cells and chloroplasts; therefore, its counts are inflated. The RAZOR EX could not measure microbial counts from edible lettuce and Mizuna leaves because they contain chloroplast DNA, but it was useful for measuring counts from edible radish bulbs, which do not contain chloroplasts (**Figure 9**).

The soilless cropping approach developed in this study represents a paradigm shift in space crop production because it provides safe-to-eat produce, eliminates discarding used substrate media after each grow-out, includes a post-harvest sanitization scheme, employs inflight methods to assess food safety, and is sustainable over multiple cropping cycles. Therefore, future spaceflight food systems can be more sustainable because they will not operate as single grow-out space biology systems, and their food safety can be verified in near-real-time using qPCR microbial monitoring methods.

## Data Availability

The raw data supporting the conclusions of this article will be made available by the authors, without undue reservation.
