# Supplementary material for: Post-harvest cleaning, sanitization, and microbial monitoring of soilless nutrient delivery systems for sustainable space crop production
Source: Front Plant Sci. 2024 Oct 11;15:1308150. doi: 10.3389/fpls.2024.1308150 (PMC11502331; doi:10.3389/fpls.2024.1308150)
Supplement: Supplementary file 1 [file Table1.docx]

**Table 1. RAZOR EX PCR cycling protocol for 16S rRNA reactions and primer combinations with TaqMan Probe.**

| Gene | Forward Primer 5’ – 3’ | Reverse Primer 5’ – 3’ | TaqMan Probe 5’ – 3’ |  |
| --- | --- | --- | --- | --- |
| *rRNA* | CGGTGAATACGTTCYCGG | GGWTACCTTGTTACGACTT | CTTGTACACACCGCCCGTC |  |
|  |  |  |  |  |
| # of Cycles | Denaturation Temperature °C | Denaturation Time (s) | Annealing Temp °C | Annealing Time (s) |
| 1 | 94 | 240 | 56 | 60 |
| 41 | 94 | 15 | 56 | 60 |
