# Supplementary material for: Post-harvest cleaning, sanitization, and microbial monitoring of soilless nutrient delivery systems for sustainable space crop production
Source: Front Plant Sci. 2024 Oct 11;15:1308150. doi: 10.3389/fpls.2024.1308150 (PMC11502331; doi:10.3389/fpls.2024.1308150)
Supplement: Supplementary file 2 [file Table2.docx]

**Table 2. Log reduction of organisms tested with chemical disinfection for down-selection.** Starting Log_10_ values are shown in parenthesis. Dashes indicate testing parameters not performed for a given organism.

| Treatments | *Time* | *P. aeruginosa* (6.00) | *S. paucimobilis* (6.43) | *B. cepacia* (5.57) | *B. pumilus* (6.29) |
| --- | --- | --- | --- | --- | --- |
| Untreated control | - | 0.07 | -0.43 | -0.43 | -0.17 |
| Ozone | 15s | 0.16 | -0.44 | 0.40 | - |
|  | 10 min. | 4.29 | 1.58 | 2.43 | - |
|  | 60 min. | 3.70 | - | - | -0.20 |
| Ozone with  3% H_2_O_2_ | 15s | 3.82 | 1.28 | 4.29 | - |
|  | 10 min. | 4.00 | 3.82 | 4.00 | - |
|  | 60 min. | 4.29 | - | - | 1.09 |
| 3% H_2_O_2_ | 15s | 4.29 | 4.29 | 4.00 | - |
|  | 10 min. | 3.82 | 4.29 | 4.00 | - |
|  | 60 min. | 4.00 | - | - | 2.18 |
| 5% ProSan | 15s | 4.00 | 4.00 | 3.82 | - |
|  | 10 min. | 3.70 | 4.00 | 4.29 | - |
|  | 60 min. | 3.82 | - | - | -0.30 |
