# Supplementary material for: Post-harvest cleaning, sanitization, and microbial monitoring of soilless nutrient delivery systems for sustainable space crop production
Source: Front Plant Sci. 2024 Oct 11;15:1308150. doi: 10.3389/fpls.2024.1308150 (PMC11502331; doi:10.3389/fpls.2024.1308150)
Supplement: Supplementary file 3 [file Table3.docx]

**Table 3. Log reduction of organisms tested with heat sterilization.** Starting Log_10_ values are shown in parenthesis. Dashes indicate testing parameters not performed with a given organism.

| 1. Treatments | Time | *P. aeruginosa* (6.00) | *B. pumilus* (6.00) |
| --- | --- | --- | --- |
| 1. Untreated control |  | -0.08 | 0.40 |
| 35 ⁰C | 15s | 0.11 | - |
|  | 2 min. | 1.00 | - |
|  | 5 min. | 0.35 | 0.16 |
|  | 10 min. | - | 0.04 |
|  | 60 min. | - | 0.14 |
| 70 ⁰C | 15s | 4.29 | - |
|  | 2 min. | 4.29 | - |
|  | 5 min. | 4.00 | 0.12 |
|  | 10 min. | 4.00 | 0.03 |
|  | 60 min. | - | 2.01 |
| 90 ⁰C | 15s | 4.00 | - |
|  | 2 min. | 4.00 | - |
|  | 5 min. | 3.82 | -0.06 |
|  | 10 min. | 3.82 | -0.05 |
|  | 60 min. | - | 0.13 |
| 90 ⁰C  3% H_2_O_2_ | 60 min. | - | 2.77 |
|  | 120 min. | - | 3.37 |
