# Supplementary material for: Post-harvest cleaning, sanitization, and microbial monitoring of soilless nutrient delivery systems for sustainable space crop production
Source: Front Plant Sci. 2024 Oct 11;15:1308150. doi: 10.3389/fpls.2024.1308150 (PMC11502331; doi:10.3389/fpls.2024.1308150)

Supplementary Material to Curry et al., 2024.

1.1 Converting RAZOR EX 16S copy number to APCs

To compare RAZOR EX values to heterotrophic plate count data, an equation was used to determine the average number of 16S copies per cell. The average number of basepairs in a 16S rRNA gene is ~1500 bp (Johnson et al. 2019). Using this number, 1 ng of E.coli DNA was calculated to contain ~ 6.083×10^8 number of 16S copies, following the equation:

Number of copies = $(ng * \frac{number}{mole})$/(basepairs * ng/g*g/mole of basepairs) (1)

This number of copies was then multiplied by 7 to approximate the number of 16S copies per E.coli cell. The following table was generated by converting 1 ng of E.coli standard to ~20 PCR cycles and 0.1 ng to ~23.3 PCR cycles:

Total 16S copies	Concentration (per 100 ul)	Number of cycles
8.69E+07	1 ng	20
8.69E+06	0.1 ng	23.3
8.69E+05	0.01 ng	26.6
8.69E+04	0.001 ng	29.9
8.69E+03	0.0001 ng	33.2
8.69E+02	0.00001 ng	36.5
8.60E+01	0.000001 ng	39.8

An “upper boundary” and “lower boundary” were determined based on the cycle number for each sample. For instance, if sample “A” has a PCR cycle number of 27, the sample would fall between 29.9 cycles and 26.6 cycles of the standard. The “lower bound” would equate to:

Lower boundary = $\left(\frac{29.9}{27}\right) * (8.69X10^4)$ (2)

and the upper boundary would be:

Upper boundary = $\left(\frac{26.6}{27}\right) * (8.69X10^5)$ (3)

These two estimates were then averaged to obtain an average number of 16S for each sample. Dilution factors were considered, and relative quantification calculated, then compared to heterotrophic plate counts.

Johnson, J.S., Spakowicz, D.J., Hong, BY. *et al.* Evaluation of 16S rRNA gene sequencing for species and strain-level microbiome analysis. *Nat Commun* **10**, 5029 (2019). https://doi.org/10.1038/s41467-019-13036-1

1.2 Time to heat 5L reservoir to temperature setpoints for various inline heaters

During heat sterilization, an inline water heater would heat 5L to a given temperature setpoint (50°C to 90°C). The table shows the time it would take to heat the 5L volume. The heating times are provided for heaters ranging from 250-750W. The data is plotted below.

Heater W	50	60	70	90
250	70	84	98	126
350	50	60	70	90
500	35	42	49	63
600	29	35	41	52
750	23	28	33	42

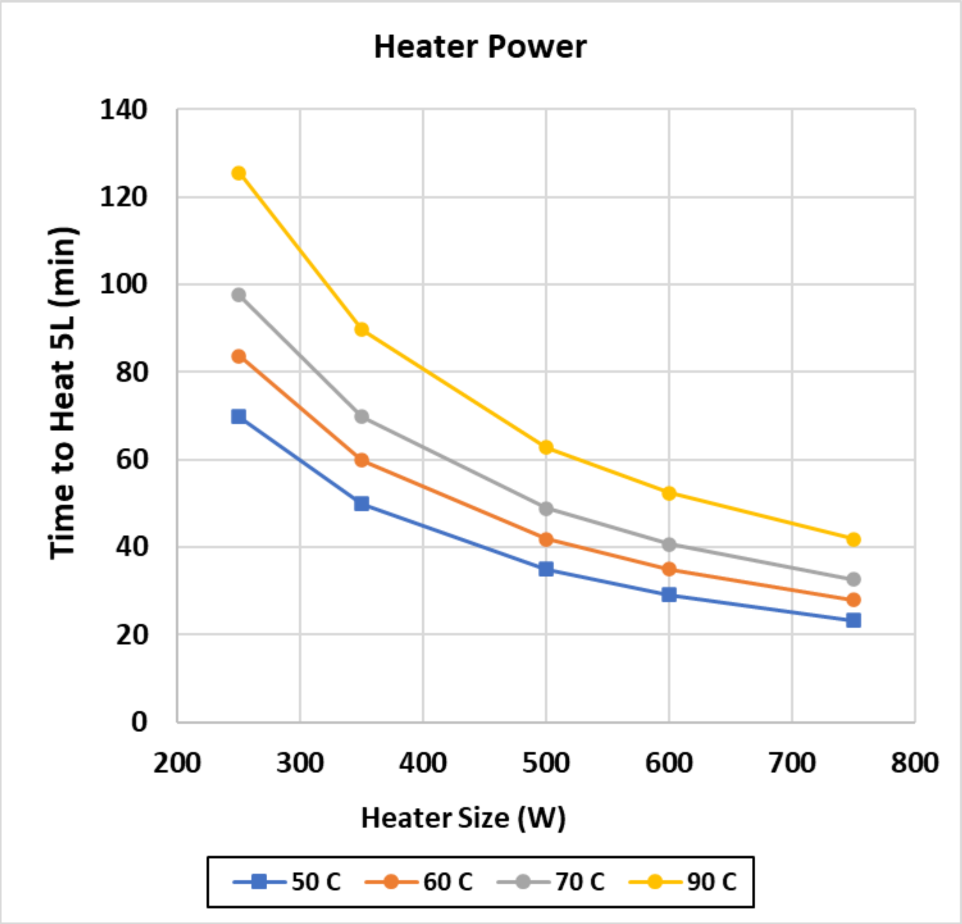

Supplement: Supplementary file 4 [file Table4.pdf]
